# Epidemiology of sports-related injuries in children and youth presenting to Canadian emergency departments from 2007–2010

**DOI:** 10.1186/2052-1847-5-30

**Published:** 2013-12-23

**Authors:** Liraz Fridman, Jessica L Fraser-Thomas, Steven R McFaull, Alison K Macpherson

**Affiliations:** 1 School of Kinesiology and Health Science, York University, 4700 Keele Street, Toronto, Ontario M3J 1P3 Canada; 2 Injury and Child Maltreatment Section, Health Surveillance and Epidemiology Division, Centre for Chronic Disease Prevention and Control, Public Health Agency of Canada, Building # 19, Tunney’s Pasture, AL 1910C Ottawa, Ontario K1A 0 K9 Canada

**Keywords:** Epidemiology, Sports-related injuries, Pediatric injuries

## Abstract

**Background:**

Although injuries related to sports and recreation represent a significant burden to children and youth, few studies have examined the descriptive epidemiology of sports-related injury since 2005, and some sports such as ringette have not been evaluated to date. The primary purpose of this study was to provide the descriptive epidemiology of sports-related injuries treated in emergency departments for children and youth aged 5 – 19.

**Methods:**

A retrospective data analysis was performed using data from the Canadian Hospitals Injury Reporting and Prevention Program [CHIRPP] from fiscal years (April – March) 2007/08 to 2009/10. CHIRPP is a computerized information system designed by the Public Health Agency of Canada that collects information about injuries to people evaluated in emergency departments across 11 pediatric hospitals and 5 general hospitals in Canada. Thirteen sports or activities were analyzed (baseball, basketball, cycling, football, ice hockey, lacrosse, ringette, rugby, skiing, sledding, snowboarding, soccer, and volleyball). Descriptive statistics, including frequency by sport, age and sex, as well as the percent of concussions within each sport were calculated.

**Results:**

Out of a total of 56, 691 reported sports and recreational injuries, soccer accounted for the largest proportion of injuries with 11,941 reported cases over the 3 year time period. Of these, approximately 30% were fractures. The 10 – 14 year age group reported the greatest proportion of injuries in 10 out of the 13 sports analyzed. In addition, males reported a greater number of overall injuries than females in 11 out of the 13 sports analyzed. The largest percentage of concussions was reported in ringette; these injuries accounted for 17.1% of overall injuries within this sport.

**Conclusions:**

Injury prevention programs in Canada should focus on improving evidence-based programs to reduce the burden of injuries in all sports.

## Background

Sports-related injuries (SRIs) are defined as injuries that occur as a result of participating in physical activity for the purposes of competition or recreation; these injuries involve individuals who participate in both organized and unorganized sports, and represent a substantial proportion of ED visits among children and youth [1-3]. Over 4 million SRIs sustained by children and adolescents are evaluated in emergency departments in the US, and between 30 and 68% of injuries sustained by children and youth are related to sport [4-6]. Finally, between 1992 and 2005, there was a reported 28% increase in the number of injuries that result from sports activity [2]. As the number of injuries resulting from sport continues to increase, the likelihood of sustaining an injury needs to be elucidated.

Findings related to sex differences in sports-related injuries are inconsistent. Several studies have reported higher injury rates for boys, but more severe injuries for girls [1,2,7]. However, it remains unclear whether males or females are more likely to sustain SRIs and how these injuries vary by sport. In the United States, football, basketball and baseball cause the greatest number of sports-related injuries in children but this varies by age, sex, and sport [5,7-10].

There is a paucity of research on pediatric SRIs in Canada. In Canada, soccer and hockey are among the most prevalent sports played by youth [11,12]. The incidence of injury by sport varies between studies however. One Canadian study reported that cycling, basketball and soccer accounted for the greatest number of reported injuries to a pediatric hospital, while another found that cycling had the highest number of injuries of any sport in Canada, accounting for 13.5% of all SRIs [3].

Overall, past research shows the nature, severity, and mechanism of pediatric injuries caused by participating in a variety of sports varies by sport, age group and sex; however, some sports such as ringette have yet to be evaluated. Further, there is a paucity of research comparing the percent of concussions to all other injuries within individual sports, and research comparing the percentage of concussions to all other injuries by sex and sport has not been reported.

The primary purpose of this study was to provide the descriptive epidemiology of SRIs treated in Canadian emergency departments for children and adolescents aged 5 – 19 from 2007/08 to 2009/10. A secondary purpose in this study was to further examine the percent of concussions in comparison to all other injuries within each sport reported by children and youth, with a focus on age and sex differences.

## Methods

### Data collection

We conducted a retrospective data analysis; data from the Canadian Hospitals Injury Reporting and Prevention Program [CHIRPP] from the 2007/08 – 2009/10 period was used for this study. CHIRPP is a computerized information system designed by the Public Health Agency of Canada that collects information about injuries to people evaluated in emergency departments across 11 pediatric hospitals and 5 general hospitals in Canada. These hospitals are located in Newfoundland, Quebec, Ontario, Manitoba, Alberta, and the Northwest Territories. When a patient arrives at the hospital the injured person or a caregiver fills out a voluntary CHIRPP form where information surrounding the circumstances of the injury are recorded including factors that contributed to the injury, the nature of the injury, body parts injured, the patient’s age and sex, as well as any treatment that was provided. The capture rate varies by hospital, and is currently between 80 – 100%. Children aged 5 to 19 who sought care in an ED between fiscal years (April – March) 2007/08 and 2009/10 were evaluated in this study. Detailed information on the CHIRPP program is available from the Public Health Agency of Canada [13].

### Study variables

The primary outcome measure in this study was any sports-related injury reported by Canadian youth and evaluated in an emergency department that participated in CHIRPP. The secondary outcome measure was the percent of concussions compared to other injuries within the same sport.

Thirteen sports were evaluated including baseball, basketball, cycling, football, ice hockey, lacrosse, ringette, rugby, skiing, sledding, snowboarding, soccer, and volleyball. These sports were chosen because they are popular sports that are coded in the CHIRPP database and some sports such as ringette have not been evaluated to date. A number of variables were analyzed in this study including age, sex, and nature of injury. Age groups were separated into three categories: 5 – 9 years, 10 – 14 years, and 15 – 19 years; these age groups were chosen because they broadly reflect developmental stages and are standardly used by the Public Health Agency of Canada [14]. Further, we examined the percent of concussions within each sport, stratified by age and sex. Using the number of other injuries within the sport as the denominator allowed us to estimate the relative percent of a concussion in each sport.

### Statistical analyses

Descriptive statistics and the percent of concussions in comparison to all other injuries within each sport were calculated. Data analyses were conducted using SPSS version 20.

### Ethics

The authors of this study were granted an exemption from ethics approval by York University as the data did not contain any identifying information and were routinely collected.

## Results

### Age differences

Between 2007 and 2010 a total of 56,691 SRIs were evaluated in Canadian emergency departments at CHIRPP hospitals. Children and adolescents aged 10 – 14 reported over half (56.6%) of the SRIs evaluated in an ED. In 11 out of the 13 sports analyzed, the 10 – 14 age group reported the greatest number of injuries; see Table 1. This was with the exception of sledding in which the 5 – 9 age group reported the greatest number of injuries and rugby where the 15 – 19 age group reported the greatest number of injuries.

**Table 1:** Age and sex distribution 2007 – 2010

**Sport/activity**	**N = 56,691 (%)**	**Age group (yr), # (%)**			**% male**
		**5-9**	**10-14**	**15-19**	
Soccer	11,941 (21.1)	1,949 (16.3)	6,946 (58.2)	3,046 (25.5)	57.7
Ice hockey	9,413 (16.6)	681 (7.2)	5,694 (60.5)	3,038 (32.3)	89.6
Cycling	8,935 (15.8)	3,009 (33.6)	4,473 (50.1)	1,453 (16.3)	73.5
Basketball	7,698 (13.6)	492 (6.4)	4,684 (60.9)	2,522 (32.8)	65.2
Football	6,141 (10.8)	388 (6.3)	3,672 (59.8)	2,081 (33.9)	91.7
Snowboarding	3,194 (5.6)	153 (4.8)	2,021 (63.3)	1,020 (31.9)	73.5
Skiing	1,970 (3.5)	470 (23.9)	1,108 (56.2)	392 (19.9)	58.4
Sledding	1,793 (3.2)	861 (48.0)	821 (45.79)	111 (6.2)	56.1
Rugby	1,651 (2.9)	8 (0.5)	376 (22.8)	1.267 (76.7)	68.4
Baseball	1,633 (2.9)	271 (16.6)	949 (58.1)	413 (25.3)	70.7
Volleyball	1,505 (2.7)	43 (2.9)	873 (58.0)	589 (39.1)	37.3
Lacrosse	493 (0.1)	26 (5.3)	291 (59.0)	176 (35.7)	85.0
Ringette	324 (0.1)	29 (9.0)	210 (64.8)	85 (26.2)	2.5

### Sex differences

In general, males reported the majority of SRIs (71.1%). Specifically, in 11 out of the 13 sports analyzed, males reported a greater number of injuries to a CHIRPP hospital than females; see Table 1. This was with the exception of volleyball and ringette where females reported a greater number of injuries.

### Sports differences

Out of a total of 56,691 reported SRIs that were evaluated in a Canadian emergency department at a CHIRPP hospital, 11,941 (21%) were sustained by youth participating in soccer; see Table 1. The sport with the lowest number of reported injuries was Ringette, which accounted for a total of 324 (0.57%) injuries.

### Nature of injury

The nature of injuries for individuals who reported a sports-related injury to a CHIRPP hospital varied by sport. In 6 out of the 13 sports analyzed, fractures accounted for the leading diagnoses among children and adolescents participating in a variety of sports; see Table 2. In basketball and volleyball, the leading diagnoses were sprains/strains. In the remaining 5 sports that included ice hockey, sledding, rugby, baseball, and ringette, the diagnoses belonged to the ‘other’ category (described elsewhere) [13]. Some of the diagnoses in this category included superficial, open wounds, fractures, dislocations, sprains/strains, injuries to nerves, blood vessels, muscle/tendons, crushing injuries etc.

**Table 2:** Nature of injury distribution, 2007 – 2010

**Sport/activity**	**Nature of injury, # (%)**				
	**Fractures**	**Sprains/strains**	**Soft tissue injuries**	**Concussions**	**Other**
Soccer	3590 (30.1)	2699 (22.6)	2628 (22.0)	413 (3.5)	2611 (21.9)
Ice hockey	2447 (26.0)	1212 (12.9)	2154 (22.9)	1023 (10.9)	2577 (27.4)
Cycling	3028 (33.9)	463 (5.2)	1334 (14.9)	356 (4.0)	2194 (24.6)
Basketball	2046 (26.6)	2246 (29.2)	1591 (20.7)	170 (2.2)	1645 (21.4)
Football	2046 (33.3)	1113 (18.1)	1274 (20.7)	345 (5.6)	1363 (22.2)
Snowboarding	1783 (55.8)	350 (11.0)	429 (13.4)	217 (6.8)	415 (13.0)
Skiing	756 (38.4)	338 (17.2)	335 (17.0)	118 (6.0)	423 (21.5)
Sledding	582 (32.5)	158 (8.8)	351 (19.6)	99 (5.5)	603 (33.7)
Rugby	391 (23.7)	259 (15.7)	328 (19.9)	182 (11.0)	491 (29.7)
Baseball	392 (24.0)	189 (11.6)	386 (23.6)	47 (2.9)	619 (37.9)
Volleyball	367 (24.4)	490 (32.6)	334 (22.2)	0 (0.0)	314 (20.9)
Lacrosse	154 (31.2)	81 (16.4)	109 (22.1)	19 (3.9)	120 (24.3)
Ringette	48 (14.8)	60 (18.5)	73 (22.5)	54 (16.7)	89 (27.5)

### Percent concussions

The greatest percent of concussions was reported in ringette; these injuries accounted for 17.1% of ED visits to CHIRPP hospitals for injuries within this sport (Table 3). Although there are variations by age group, in general girls sustained a higher percentage of concussions in 6 out of the 13 sports analyzed. Males had higher percentages of concussions than females in basketball, cycling, football, lacrosse, sledding and snowboarding (Table 4). Boys and girls had a similar percentage of concussions in skiing. When age and sex were included, female children aged 5 – 9 who participated in ringette reported the greatest percentage of concussions (20.7%) in comparison to all other injuries within this sport. Males aged 10 – 14 who played hockey sustained the greatest percentage of concussions among males of which concussions accounted for 11.4% of all hockey injuries in that age group. The lowest percentage of concussions was seen in children and youth who played volleyball (1.9%).

**Table 3:** Percent concussion distribution for females 2007 – 2010

**Sport/activity**	**Age group (yr), # (%)**			**% of concussions all ages**
	**5-9**	**10-14**	**15-19**	
Ringette	6 (20.7)	36 (17.5)	12 (14.8)	17.1
Ice hockey	*	74 (12.5)	52 (16.3)	13.3
Rugby	0 (0.0)	11 (14.5)	53 (11.9)	12.3
Snowboarding	*	21 (4.4)	24 (7.1)	5.5
Skiing	5 (2.4)	24 (5.2)	20 (13.6)	6.0
Football	0 (0.0)	12 (3.8)	6 (3.7)	3.5
Sledding	13 (3.5)	17 (4.8)	6 (10.2)	4.6
Cycling	35 (3.2)	40 (3.8)	8 (3.7)	3.5
Lacrosse	*	0 (0.0)	0 (0.0)	2.7
Soccer	8 (1.5)	96 (3.2)	107 (7.0)	4.2
Baseball	*	18 (6.6)	*	4.6
Basketball	*	33 (1.8)	23 (3.2)	2.1
Volleyball	0 (0.0)	15 (2.6)	5 (1.5)	2.1

*Cell size <5.

**Table 4:** Percent concussion distribution for males 2007 – 2010

**Sport/activity**	**Age Group (yr), # (%)**			**% of concussions all ages**
	**5-9**	**10-14**	**15-19**	
Ringette	**	**	**	0.0
Ice Hockey	56 (9.1)	580 (11.4)	257 (9.5)	10.6
Rugby	0 (0.0)	30 (10.0)	87 (10.6)	10.4
Snowboarding	6 (6.7)	89 (6.4)	76 (8.7)	7.3
Skiing	12 (4.6)	40 (6.2)	17 (6.9)	6.0
Football	9 (2.5)	178 (5.3)	140 (7.3)	5.8
Sledding	36 (3.5)	17 (4.8)	*	6.3
Cycling	54 (2.8)	152 (4.4)	67 (5.4)	4.2
Lacrosse	*	13 (5.2)	*	4.1
Soccer	39 (2.7)	110 (2.8)	53 (3.5)	2.9
Baseball	*	15 (2.2)	6 (2.3)	2.2
Basketball	6 (1.6)	76 (2.7)	31 (1.7)	2.3
Volleyball	0 (0.0)	7 (2.3)	*	1.4

*Cell size <5.

**No males sustained a concussion playing ringette.

## Discussion

During the three-year study period between 2007–2010 some general trends were apparent within the injury data across sports. First, youth participating in soccer accounted for the greatest number of sports-related injuries evaluated in an emergency department. As previously noted, soccer is the most popular sport in Canada, which could partially explain the high number of reported injuries in this sport [12]. Further, given that soccer is a sport that requires minimal equipment, more children may participate in unorganized soccer than in other sports that require specialized equipment and sporting environment such as hockey, skiing and snowboarding. The high number of children and youth who are injured while playing soccer may be sustaining injuries during both organized and unorganized play.

In general, males sustained a greater number of overall injuries than females in every sport except for volleyball and ringette. In addition children participating in soccer, sledding, and skiing reported nearly an equal number of injuries irrespective of sex. A study performed by Pakzad-Vaezi in 2011 reported similar findings; in their study males accounted for 68% of the SRIs reported to emergency departments [2].

The highest percentage of concussions was reported among females between the ages of 5 and 9 participating in ringette. Throughout the literature there was a paucity of research comparing the percentage of concussions sustained by males and females of different ages within each sport. The findings in this study are consistent with Randazzo et al. who reported that girls were more likely to sustain a TBI [7]. This was the first study to report on the percentage of concussions seen in ringette and the findings show that young girls are reporting the greatest number of concussions in comparison to all other injuries within this sport.

Youth between the ages of 10 and 14 reported the greatest number of injuries in every sport except for sledding where children aged 5–9 reported the majority of injuries and rugby where adolescents aged 15–19 reported the majority of injuries. These findings are consistent with the literature on SRIs. Several studies have demonstrated that youth aged 10–14 most frequently report a SRI to the emergency department [1,3,4,15,16]. The Public Health Agency of Canada reports that 68% of SRIs occur to children aged 10–14 [6].

Several studies that have examined the nature of sport-related injuries among youth reported that fractures were most frequently evaluated in the emergency departments [1,5,10,17]. A study performed by Taylor and Attia in 2000 showed that fractures accounted for 29.4% of all sports-related diagnoses [15]. Our study was consistent with the literature as fractures were the most common diagnosis in 6 out of the 13 sports, with the exception of volleyball and basketball where sprains/strains were more frequently diagnosed, and ice hockey, sledding, rugby, baseball, and ringette where ‘other’ injuries were most frequently diagnosed.

The main strength of this study is that our findings are generalizable to Canadians because we drew upon data from many provinces [18]. The main limitation of this study is that we were only able to capture youth who were evaluated in an emergency department with the CHIRPP surveillance system for a SRI. Unfortunately our study could not account for children and adolescents who were seen in other emergency departments or by a family physician, chiropractor, sports therapist, etc. Therefore the number of sustained injuries over this time period was certainly higher than the statistics presented here, because children and youth clearly visit other EDs and sources of care. Data on CHIRPP’s validity and limitations have been published elsewhere [19]. In addition, because CHIRPP uses self-reported data this may have introduced a recall bias into our study. Finally, without knowing how many youth participated in formal and informal sport and physical activity within this time period, we could not calculate rates of injury in this study. Future studies should focus on obtaining participation rates in order to be able to evaluate a truer measure of sports risk.

## Conclusions

Injuries sustained by youth in Canada differ greatly by age, sex, and sport discipline. In Canada, future injury prevention should focus on youth participating in soccer as it showed the greatest amount of SRIs. In addition, injury prevention policies should also target children aged 10–14. Although males are injured most frequently in the majority of sports females suffer from a higher percentage of concussions in comparison to all other injuries in some sports and therefore preventing concussions among females in sports such as ringette should be a priority. Concussion prevention strategies need to take age and gender into account. The need to incorporate evidence-based strategies and policies that reduce injuries is reflected in these data.

## Abbreviations

SRI: Sports-related injuries; ED: Emergency department; CCHS: Canadian community health survey; PHAC: Public health agency of Canada; CHIRPP: Canadian hospitals injury reporting and prevention program; TBI: Traumatic brain injury.

## Competing interests

The authors of this study declare that they have no financial or non-financial competing interests.

## Authors’ contributions

LF performed the statistical analyses and drafted the manuscript for this study. JLFT made critical revisions to the manuscript. SRM helped in the acquisition and organization of data. AKM aided in designing the study and interpreting the data. All authors read and approved the final manuscript.

## Pre-publication history

The pre-publication history for this paper can be accessed here:

http://www.biomedcentral.com/2052-1847/5/30/prepub
